# Maxillary sinus volume and septa morphology in relation to dentition status: a cone-beam computed tomography–based three-dimensional analysis

**DOI:** 10.1186/s12903-026-08000-7

**Published:** 2026-02-27

**Authors:** Rabia Atay, Melda Mısırlıoğlu

**Affiliations:** Department of Oral and Maxillofacial Radiology, Faculty of Dentistry, Kırıkkale University Yahşihan, Kırıkkale, 71450 Turkey

**Keywords:** Cone-beam computed tomography, Maxillary sinus, Dentition, Edentulous, Three-dimensional analysis

## Abstract

**Background:**

The maxillary sinus shows considerable anatomical variation influenced by dentition status and craniofacial factors. Understanding these variations is essential for surgical planning and implant dentistry. This study aimed to evaluate maxillary sinus volume and septa morphology in relation to dentition status, age, and sex using cone-beam computed tomography (CBCT) and semi-automated segmentation.

**Methods:**

CBCT scans of 84 patients (168 maxillary sinuses) were retrospectively analyzed. Sinus volumes were measured using ITK-SNAP, while septa presence, location, orientation, and height were assessed on multiplanar reformatted images. Patients were categorized as dentate, partially dentate, or edentulous. Statistical analyses were performed using appropriate parametric and non-parametric tests, with significance set at *p* < 0.05.

**Results:**

Total maxillary sinus volume differed significantly according to dentition status, with the smallest volumes observed in edentulous individuals (*p* < 0.001). Male patients exhibited significantly larger sinus volumes than females bilaterally (*p* < 0.05). Septa were most frequently located in the middle region and predominantly exhibited a coronal orientation. Septa presence showed a significant association with dentition status on the left side (*p* < 0.05), whereas septa height did not differ significantly among dentition groups. No significant correlations were found between septa characteristics and age or sex.

**Conclusion:**

Maxillary sinus volume is strongly influenced by dentition status and sex, while septa prevalence shows a partial association with dental status. Semi-automated CBCT-based volumetric analysis provides a reliable method for assessing sinus anatomy and may support safer and more predictable outcomes in sinus augmentation and implant-related procedures.

**Supplementary Information:**

The online version contains supplementary material available at 10.1186/s12903-026-08000-7.

## Introduction

The maxillary sinuses are paired, pyramid-shaped, epithelium-lined air cavities located within the maxilla, lateral to the nasal cavity and inferior to the orbits [1]. Owing to their close anatomical relationship with the oral cavity, maxillary sinuses are clinically relevant—particularly in implant-related procedures and sinus augmentation, where anatomical variations may influence surgical planning and outcomes. Beyond its clinical relevance, maxillary sinus morphology—particularly sinus volume—has also been reported to have forensic applications, such as sex determination [2].

The maxillary sinus begins to develop in utero and continues postnatally, reaching adult dimensions around the age of 18, typically following the eruption of permanent dentition [3]. Sinus morphology and volume are influenced by multiple factors, such as age, sex, dentition status, local pathological conditions, genetic predisposition, and variations in adjacent nasal structures [4].

Among the internal anatomical features, maxillary sinus septa are thin cortical bony partitions arising from the sinus floor or lateral walls that divide the cavity into compartments. Maxillary sinus septa are of particular surgical importance because they may complicate sinus floor elevation and increase the risk of Schneiderian membrane perforation; therefore, accurate preoperative identification and characterization are essential [5, 6].

Earlier investigations of maxillary sinus anatomy and volume were largely limited to cadaveric studies or two-dimensional imaging techniques, such as lateral cephalometric and posteroanterior radiographs [7]. The advent of CBCT has substantially improved sinus assessment by providing high-resolution, three-dimensional visualization with a relatively low radiation dose [8]. In parallel, the development of semi-automated segmentation software—such as Mimics, Dolphin, OsiriX, and ITK-SNAP—has enabled precise reconstruction and volumetric analysis of anatomical structures, with reported error rates below 2% [9]. Together, these advances have made CBCT-based three-dimensional analysis a reliable and widely accepted approach for quantitative evaluation of paranasal and craniofacial anatomy in both clinical and research settings.

Several previous studies have evaluated maxillary sinus volume under different conditions and in various populations. However, many of these studies primarily focused on isolated parameters, such as age, sex, or dentition status, while comprehensive three-dimensional assessments simultaneously integrating sinus volume, septa morphology, and dentition status remain limited. Therefore, the present study aimed to comprehensively evaluate variations in maxillary sinus volume according to dentition status (dentate, partially dentate, and edentulous), age, and sex using CBCT imaging and semi-automated segmentation in ITK-SNAP. In addition, the study assessed the prevalence, orientation, location, and length of maxillary sinus septa, and explored their potential associations with demographic and anatomical variables. We hypothesized that maxillary sinus volume and septa morphology may be associated with dentition status.

## Materials and methods

### Study design and ethical approval

This retrospective study was conducted in accordance with the principles of the Declaration of Helsinki and was approved by the Non-Interventional Research Ethics Committee of Kirikkale University. The study utilized CBCT images from the patient archive of the Department of Oral, Dental, and Maxillofacial Radiology, Faculty of Dentistry, Kirikkale University.

### Study population

CBCT scans obtained between 2021 and 2024 were retrospectively reviewed. The study included patients aged 18 years or older whose scans provided complete visualization of both maxillary sinuses without evidence of sinus pathology or anatomical anomalies. Patients with a history of sinus surgery or trauma, those younger than 18 years, and scans with poor image quality were excluded.

A total of 84 patients (42 males and 42 females) were included. Based on posterior maxillary dentition, participants were categorized as dentate (*n* = 28), partially dentate (*n* = 28), or edentulous (*n* = 28). Each right and left sinus was evaluated separately, yielding 168 volumetric measurements.

### CBCT imaging protocol

All CBCT scans were obtained using an I-CAT device (Imaging Sciences, Hatfield, PA, USA). Patients were positioned with the Frankfurt horizontal plane parallel to the floor. Scanning parameters included a field of view (FOV) of 8 × 15 cm, 15 × 21 cm, or 21 × 21 cm; tube voltage of 120 kVp; tube current of 8 mA; an exposure time of 8.9 s; and a voxel size of 0.3 mm. DICOM data sets were exported for further analysis.

### Volumetric analysis

**Fig. 1:**
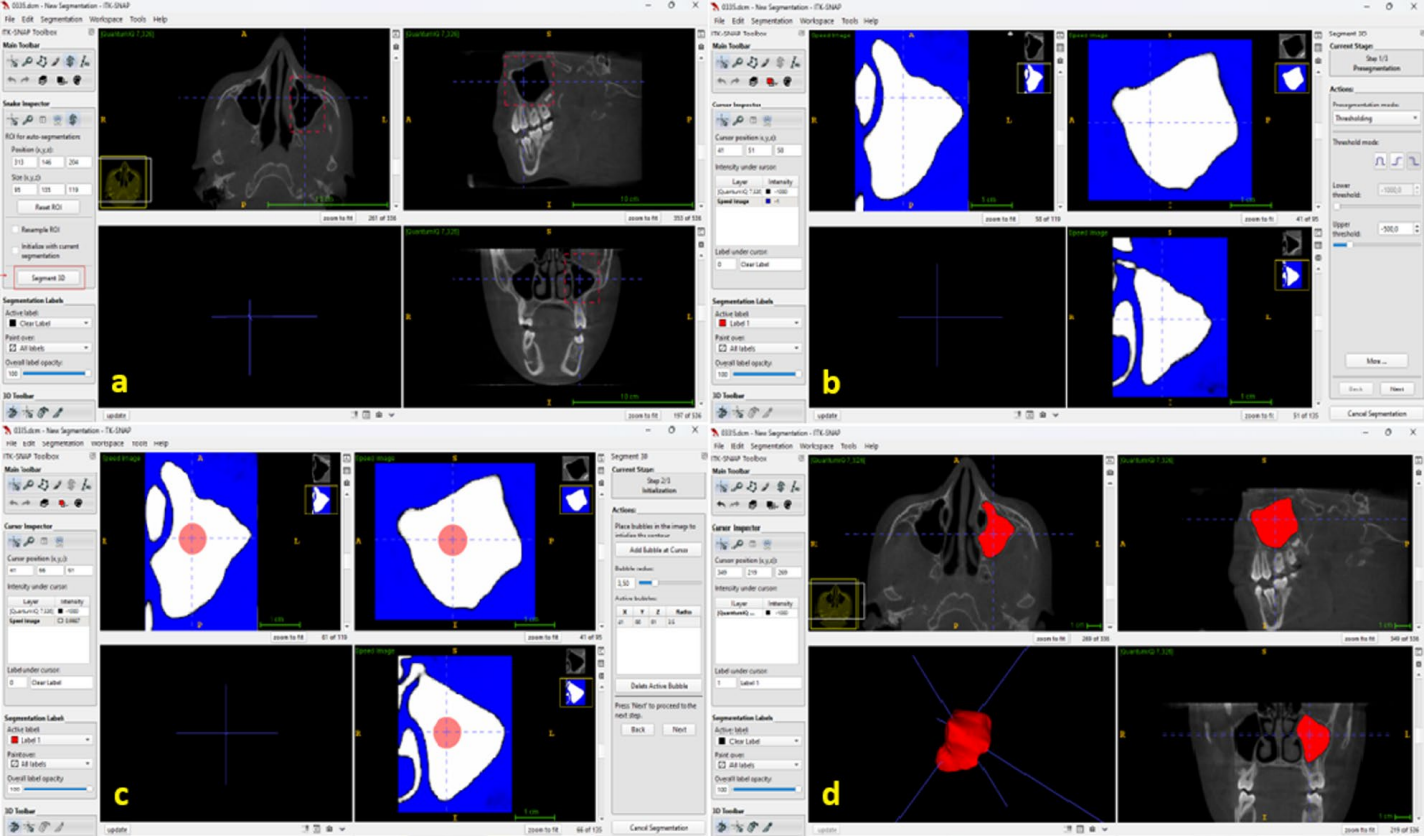
Measurement of maxillary sinus volume using ITK-SNAP. **a **Coronal, sagittal, and axial views of the left maxillary sinus delineated in a rectangular region. **b **Visualization of the maxillary sinus with threshold values set between − 1000 and − 500. **c **Determination of the seed point for semi-automated maxillary sinus segmentation. **d **Axial, sagittal, coronal, and three-dimensional views of the maxillary sinus

DICOM files were imported into ITK-SNAP software (version 4.0.2) for volumetric evaluation. For each sinus, a rectangular region of interest (ROI) was defined (Fig. 1a), and semi-automated segmentation was performed using the Active Contour tool based on voxel-intensity thresholding, with a lower threshold of − 1000 and an upper threshold of − 500 to isolate the air-filled sinus cavity (Fig. 1b). This threshold window was selected to include the aerated sinus lumen while excluding surrounding bone and soft tissues. Seed points were placed within each sinus, and a consistent segmentation workflow—including ROI placement, seed-point initialization, and Active Contour evolution—was applied to all cases. The resulting segmentations were reviewed in axial, coronal, and sagittal planes, and minor manual refinements were performed when necessary to achieve anatomically accurate delineation of the sinus boundaries (Fig. 1c, d). Final sinus volumes were automatically calculated and recorded in cubic millimeters (mm³).

### Septa evaluation

The number, location, height, and orientation of maxillary sinus septa were evaluated using Invivo Dental software (Anatomage, San Jose, CA, USA). Septal height was measured from the sinus floor to the apex. The anatomical location of each septum was classified as anterior (from the anterior wall to the second premolar), middle (between the first and second molars), or posterior (from the distal aspect of the second molar to the posterior wall), following the method described by Kim et al. [10]. Septal orientation was categorized as coronal (buccopalatal), sagittal, or transverse (horizontal). All volumetric and septa measurements were performed by a single trained oral and maxillofacial radiology researcher (R.A.) with two years of experience in CBCT-based three-dimensional image analysis.

### Statistical analysis

An a priori power analysis was performed using G*Power software (version 3.1.9.2) for a one-way ANOVA with three groups. Assuming a large effect size (f = 0.40), an alpha level of 0.05, and a target power of 85%, the required sample size was calculated as 81 patients. As 84 patients were included in the present study, the sample size was considered statistically sufficient. All statistical analyses were performed using IBM SPSS Statistics (version 25.0; IBM Corp., Armonk, NY, USA). Data normality was assessed using the Shapiro–Wilk test. Depending on data distribution, independent samples t-test, Mann–Whitney U test, or Kruskal–Wallis test were applied for group comparisons. Spearman’s correlation coefficient was used for continuous non-parametric data. Categorical variables were analyzed using Pearson’s chi-square test or Fisher’s exact test. Because the right and left maxillary sinuses belong to the same individual, bilateral measurements were treated as paired data and were compared using the Wilcoxon signed-rank test. Intra-observer reliability was assessed by re-evaluating 25% of randomly selected CBCT images using kappa statistics for categorical variables and intraclass correlation coefficients (ICC) for continuous measurements. A p-value < 0.05 was considered statistically significant.

## Results

A total of 168 maxillary sinuses from 84 patients (42 males and 42 females; mean age ± SD: 48.26 ± 15.79 years; range: 18–80 years) were evaluated. Edentulous patients exhibited significantly lower sinus volumes compared with the dentate and partially dentate groups (*p* < 0.05), with mean volumes of 12.27 ± 4.75 cm³ for edentulous, 14.91 ± 4.11 cm³ for partially dentate, and 15.83 ± 5.62 cm³ for dentate individuals (Table 1).

**Table 1:** Distribution and comparison of maxillary sinus volumes according to dentition status

	**DentitionStatus**	**Min-Max(cm** ^**3**^ **)**	**IQR (25th–75th)**	**Mean ± SD (Median)**	**TestStatistic**	***p*** **-value**
Right	Partially	7.13–22.41.13.41	12.2–16.47.2.47	14.51±3.53 (14.58)	7.322	0.026*
	Dentate	8.3–31.83.3.83	12.68–19.5	15.91 ± 5.98 (14.38)		
	Edentulous	4.21–22.8	8.39–15.8	12.13 ± 5.02 (10.92)		
Left	Partially dentate	6.54–27.64	11.11–18.61	15.31 ± 4.65 (15.57)	7.454	0.024*
	Dentate	7.11–29.51	11.79–18.83	15.74 ± 5.34 (14.48)		
	Edentulous	5.56–19.99	8.86–17.06	12.41 ± 4.55 (11.25)		
Total	Partially dentate	6.54–27.64	11.82–17.4	14.91 ± 4.11 (15.27)	15.253	< 0.001*
	Dentate	7.11–31.83	11.82–19.24	15.83 ± 5.62 (14.43)		
	Edentulous	4.21–22.8	8.86–15.92	12.27 ± 4.75 (11.03)		

* Values are expressed as mean ± SD (median); Kruskal–Wallis test with Bonferroni correction was used (*p* < 0.05)

Male patients consistently demonstrated larger sinus volumes than females (15.58 ± 5.94 cm³ vs. 12.98 ± 3.64 cm³; *p* < 0.05) (Table 2).

**Table 2:** Distribution and comparison of maxillary sinus volumes according to gender

	**Gender**	**Min-Max(cm** ^3^ **)**	**Interquartile Range**	**Mean ± SD**	**Test Statistic**	**p-value**
Right	Female	4.55–19.66.55.66	10.69–15.32.69.32	12.82±3.51 (12.79)	−2.464	0.016*
	Male	4.21–31.83	11.31–20.16	15.45 ± 6.16 (14.72)		
Left	Female	5.56–21.77	10.54–15.88	13.14 ± 3.81 (11.98)	−2.469	0.016*
	Male	5.88–29.51	11.06–18.89	15.72 ± 5.78 (16.32)		
Total	Female	4.55–21.77	10.69–15.37	12.98 ± 3.64 (12.56)	−3.506	0.001*
	Male	4.21–31.83	11.15–19.21	15.58 ± 5.94 (15.51)		

* Values are expressed as mean ± SD (median); independent samples *t*-test was used (*p* < 0.05)

**Fig. 2:**
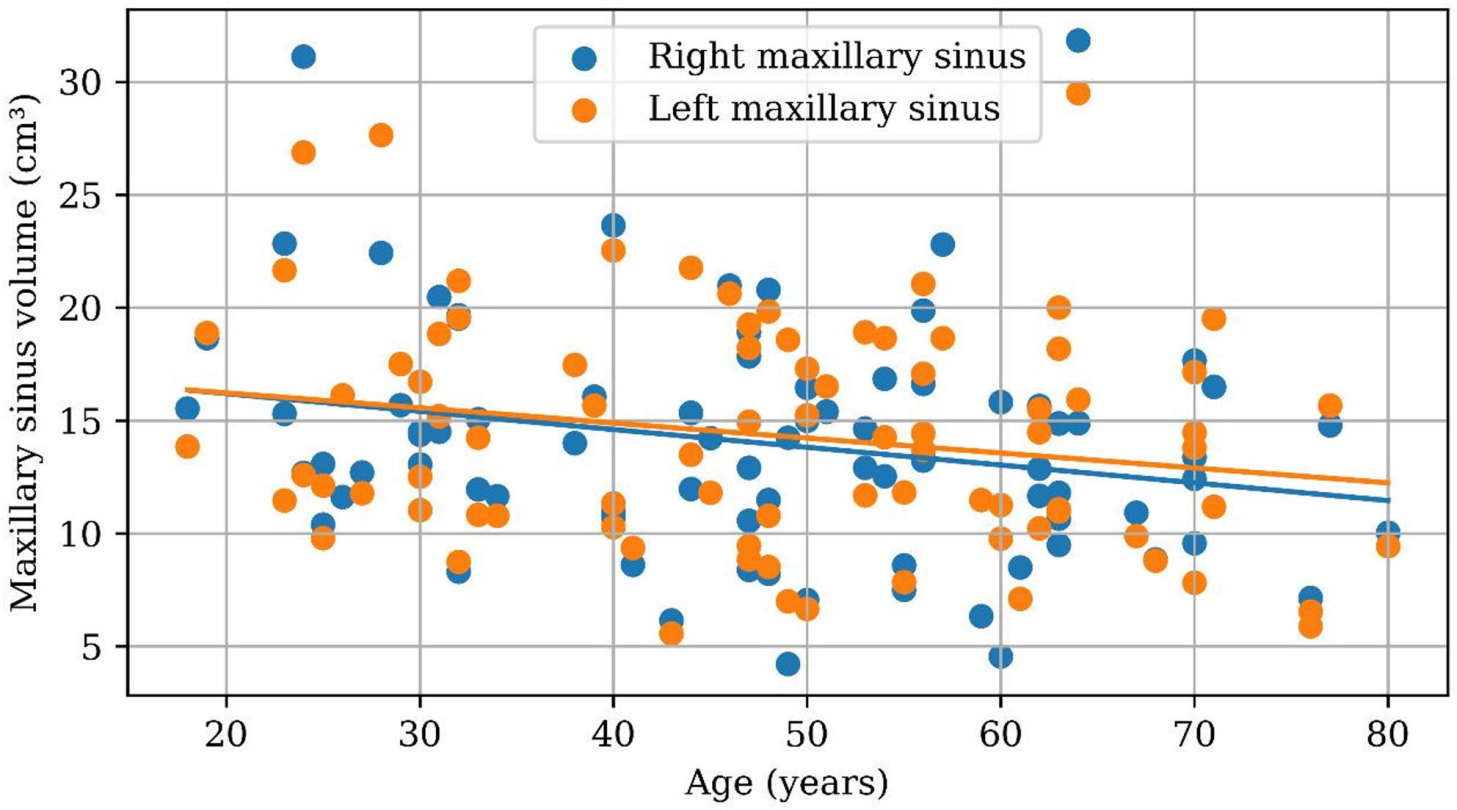
Scatter plot showing the relationship between age and maxillary sinus volume for the right and left sinuses. No significant volumetric difference was found between the right and left sinuses (Wilcoxon signed-rank test, *p* = 0.686)

Age showed a weak but significant negative correlation with sinus volume (right: *r* = − 0.245, *p* = 0.021; left: *r* = − 0.218, *p* = 0.041), indicating a gradual decrease in sinus size with advancing age (Table 3) (Fig. 2).

**Table 3:** The relationship between age and maxillary sinus volume measurement

	**r**	**p**
Right maxillary sinus volume	−0.245	0.021*
Left maxillary sinus volume	−0.218	0.041*

Spearman’s correlation analysis was used (*p* < 0.05)

Maxillary sinus septa were most prevalent among edentulous patients, particularly on the left side (57.1%), whereas partially dentate and dentate individuals exhibited lower prevalence rates (Table 4). Most septa were located in the middle region of the sinus, although additional posterior septa were occasionally observed in partially dentate patients.

**Table 4:** Distribution of septa prevalence according to dentition status

	**Dentition**	**n**	**%**	**%P.**	**n**	**%**	**%P.**	**Test Statistic**	**p**
Right	Partially dentate	1	67.9	36.5	9	32.1	28.1	2.524	0.283
	Dentate	19	67.9	36.5	9	32.1	28.1		
	Edentulous	14	50.0	26.9	14	50.0	43.8		
Left	Partially dentate	25	89.3	39.7	3	10.7	14.3	7.647	0.030*
	Dentate	22	78.6	34.9	6	21.4	28.6		
	Edentulous	16	57.1	25.4	12	42.9	57.1		

*Data are presented as number and percentage; comparisons were performed using the chi-square test (*p* < 0.05)

In terms of orientation, coronal septa were dominant (70.9%), followed by transverse (19.3%) and sagittal (9.8%) orientations (Table 5). The mean septal height across the sample was 7.75 mm, with no significant variation by sex, dentition status, or orientation.

Intraobserver agreement analysis demonstrated good to excellent reliability. Kappa statistics were > 0.61, and intraclass correlation coefficients (ICC) were > 0.70 for all measured variables, indicating consistent and reproducible measurements.

**Table 5:** Distribution and association of septa orientations according to dentition status

			**Partially Dentate**	**Dentate**					**Edentulous**									
					n	%		%D.		n	%		%D.	n	%	%D.	Test Statistic	p
Right		Coronal			5	18.5		55.6		7	25.9		70.0	15	55.6	83.3	4.016	0.404
		Transversal			3	50.0		33.3		1	16.7		10.0	2	33.3	11.1		
		Sagittal			1	25.0		11.1		2	50.0		20.0	1	25.0	5.6		
Left		Coronal			0	0.0		0.0		6	35.3		85.7	11	64.7	73.3	10.184	0.011*
		Transversal			1	16.7		33.3		1	16.7		14.3	4	66.7	26.7		
		Sagittal			2	100.0		66.7		0	0.0		0.0	0	0.0	0.0		

* Data are presented as number and percentage; associations were analyzed using the chi-square test (*p* < 0.05)

These findings suggest that dentition status, sex, and age may influence maxillary sinus morphology and septa prevalence, which should be considered during preoperative assessment and surgical planning for sinus augmentation and implant-related procedures.

## Discussion

The present study comprehensively evaluated maxillary sinus volume and septal characteristics in relation to dentition status, sex, and age using CBCT and ITK-SNAP software. The findings revealed several clinically and anatomically relevant patterns that are largely consistent with previous literature.

The maxillary sinus is an anatomically and clinically significant structure in dental practice due to its close relationship with the posterior maxillary teeth and alveolar ridge [2]. Understanding its morphology, volume, and internal architecture is essential for procedures such as sinus floor elevation, implant placement, orthognathic surgery, and endodontic treatment involving the posterior maxilla [11, 12]. Furthermore, the use of sinus volume in forensic sex determination has gained interest because of its anatomical resilience and measurable sexual dimorphism [13].

Our results demonstrated that edentulous patients exhibited significantly reduced sinus volumes compared with dentate and partially dentate individuals. This observation aligns with earlier studies attributing the reduction to the loss of mechanical stimulation after tooth extraction and subsequent alveolar bone resorption [1, 14]. Yamaguchi et al. [15], using CBCT and Mimics software, reported that pneumatization is significantly reduced in areas lacking posterior teeth. Similarly, Martínez-González et al. [16] found greater sinus volumes in patients with fewer missing teeth. In contrast, Bornstein et al. [17] and Luz et al. [18] did not observe significant volumetric differences; however, the young cohort in Bornstein et al. (mean age 29.5 years) and the low number of edentulous patients in Luz et al. (*n* = 9) may have limited detection of dentition-related volume differences.

With respect to sex, males exhibited significantly larger sinus volumes than females, consistent with previous findings [2, 19, 20]. Ekizoglu et al. [21] reported over 77% accuracy for sex estimation based on sinus volume in the Turkish population, emphasizing its forensic value. These differences likely reflect sexual dimorphism in craniofacial morphology. However, Ariji et al. [22], who used conventional CT, did not detect significant sex-related differences, which may be due to differences in imaging resolution and analytic protocols.

A weak but significant negative correlation was found between age and sinus volume, indicating that sinus size tends to decline with increasing age. This finding is consistent with Iwai et al. [23], who observed a reduction in sinus volume after the fifth decade of life. Jun et al. [24] noted that this reduction occurs earlier in females (in their 20 s) compared with males (in their 30 s), possibly related to hormonal or skeletal maturation differences. Kumar et al. [25] similarly reported a gradual post-adolescent decline beginning around ages 17–26. In contrast, Martínez et al. [16] found no age-related differences, likely due to their narrower age range (40–76 years). The broad age range (18–80 years) in the present study may have provided greater sensitivity to detect such changes.

No significant volumetric difference was detected between the right and left sinuses, supporting previous CBCT-based studies by Gülec et al. [26] and Toprak et al. [6]. This symmetry suggests that sinus development is typically bilateral and uniform in the absence of pathology or trauma.

In terms of maxillary sinus septa, the present study observed a higher prevalence in edentulous patients, consistent with Kim et al. [10] and Krennmair et al. [27], who attributed this to compensatory bone remodeling. Koymen et al. [28] reported similar findings, whereas Schriber et al. [29] found no dentition-related variation. The prevalence values in this study (38% right, 25% left) correspond closely with the ranges reported by Malec et al. [30], who found an average global septa prevalence of 33.2%.

No significant association was identified between septa prevalence and sex or sinus volume, which aligns with the findings of Neychev et al. [31]. Conversely, Park et al. [32] reported higher prevalence in females, while Koymen et al. [28] and others [33] observed a higher prevalence in males. These discrepancies may result from population-specific differences or variations in defining primary versus secondary septa.

Septa were predominantly located in the middle region, consistent with Kim et al. [10], Valesquez-Plata et al. [34], and Malec et al. [30]. A posterior shift was observed in partially dentate and edentulous patients, which may be explained by the formation of secondary septa following posterior tooth loss. In contrast to primary septa, which are developmental and typically show a more regular and symmetric distribution, secondary septa arise from irregular pneumatization and alveolar bone remodeling and therefore tend to be more variable in location and morphology. Clinically, this posterior clustering of secondary septa increases the risk of membrane perforation during sinus floor elevation and should be carefully evaluated on preoperative CBCT [10, 27].

Regarding orientation, coronal (buccopalatal) septa were most prevalent, followed by transverse and sagittal orientations. This pattern corresponds to the findings of Wang et al. [35], Pommer et al. [36], and Toprak et al. [6], all of whom reported coronal dominance in over 70% of cases. Additionally, the present study found a strong bilateral correlation in septal orientation, indicating anatomical consistency across sides.

The mean septal height of 7.75 mm observed in this study is within the range reported in previous studies (2.5–12.7 mm). Underwood first described this variation in 1910, and more recent data from Henriques et al. [37] and Pommer et al. [36] reported mean values of 6.3 mm and 6.9 mm, respectively. Although slightly higher mean values were observed in dentate patients (8.75 mm), the difference was not statistically significant, likely reflecting the predominance of primary septa formed during sinus development rather than secondary septa arising after tooth loss.

### Study limitations

A key limitation of this retrospective design is the potential confounding between chronological age and dentition status, as tooth loss becomes more prevalent with advancing age. Therefore, part of the observed age-related decrease in maxillary sinus volume may reflect dentition-related remodeling rather than aging alone. Ideally, age-matched comparisons between dentate and edentulous individuals would be required to disentangle these effects; however, such matching is challenging in routine clinical archives because fully edentulous young adults and fully dentate older adults are uncommon. Accordingly, the age–volume association in the present study should be interpreted in conjunction with dentition status, and future studies with age-matched groups or multivariable modeling are warranted to clarify the independent contribution of age. In addition, although CBCT acquisitions were standardized, gray-value variability inherent to CBCT imaging may still influence tissue contrast and segmentation boundaries.

### Significance of the study

The clinical relevance of the present findings lies in the direct impact of dentition status on maxillary sinus volume and septal morphology, both of which are critical determinants in implant planning and sinus augmentation procedures. The significantly reduced sinus volumes observed in edentulous patients indicate advanced alveolar bone resorption and altered sinus pneumatization, conditions that increase the complexity and risk of sinus floor elevation and implant placement. Moreover, the higher prevalence and posterior shift of septa in partially dentate patients suggest a greater likelihood of intraoperative membrane perforation during lateral window or transcrestal sinus lift procedures. Therefore, three-dimensional CBCT-based volumetric and septal evaluation provides essential preoperative information for selecting the appropriate surgical approach, determining graft volume, and minimizing complications. From a scientific perspective, this study is among the few to combine dentition status, volumetric sinus assessment, and detailed septa characterization using standardized semi-automated segmentation with ITK-SNAP. By demonstrating that both sinus volume and septal patterns vary systematically according to dental status, age, and sex, the present findings contribute to a more refined anatomical understanding of maxillary sinus remodeling. This integrated three-dimensional framework provides reference data for future morphometric, clinical, and forensic studies and supports the use of CBCT-based volumetric analysis as a reproducible tool in craniofacial research.

## Conclusion

This study demonstrates that maxillary sinus anatomy varies systematically according to dentition status, sex, and age, with clinically relevant differences in sinus volume and internal architecture. By employing semi-automated three-dimensional segmentation with ITK-SNAP, the present study provides reliable volumetric data that support individualized anatomical assessment.

From a clinical perspective, the reduced sinus volume and increased septa prevalence observed in edentulous patients indicate a higher likelihood of complex sinus anatomy, which may complicate sinus floor elevation and implant placement. Routine CBCT-based volumetric and septal evaluation can therefore assist in selecting appropriate surgical approaches, reducing the risk of Schneiderian membrane perforation, and improving the safety and predictability of implant and sinus augmentation procedures.

## Supplementary Information

Supplementary Material 1

## Data Availability

The datasets generated and/or analyzed during the current study are available from the corresponding author upon reasonable request.

## Abbreviations

CBCT: Cone Beam Computed Tomography
3D: Three-Dimensional
