# Supplementary material for: Maxillary sinus volume and septa morphology in relation to dentition status: a cone-beam computed tomography–based three-dimensional analysis
Source: BMC Oral Health. 2026 Feb 27;26:586. doi: 10.1186/s12903-026-08000-7 (PMC13049738; doi:10.1186/s12903-026-08000-7)
Supplement: Supplementary file 1 — Supplementary Material 1 [file 12903_2026_8000_MOESM1_ESM.pdf]

KIRIKKALE ÜNİVERSİTESİ

GİRİŞİMSEL OLMAYAN ARAŞTIRMALAR ETİK KURUL KARARI

Toplantı Tarihi: 19/04/2023
Toplantı Sayısı: 2023/04
Karar No: 2023.04.16

Kırıkkale Üniversitesi Girişimsel Olmayan Etik Kurulu 19/04/2023 tarihinde çarşamba günü saat 10.00'da Prof. Dr. Sema ZERGEROĞLU'nun başkanlığında toplanmıştır.

Kırıkkale Üniversitesi Diş Hekimliği Fakültesi Ağız Diş ve Çene Radyolojisi Anabilim Dalı, Doç. Dr. Melda MISIRLIOĞLU'nun danışmanlığında yürütülecek olan Arş. Gör. Rabia ÇELİK'in uzmanlık tezi **"Dişli, Dişsiz ve Kısmi Dişli Hastalarda Maksiller Sinüs Hacmi ve Septa Prevelansının Konik Işınlı Bilgisayarlı Tomografi ile Değerlendirilmesi"** isimli başvurusu Kırıkkale Üniversitesi Girişimsel Olmayan Araştırmalar Etik Kurulu Yönergesinde belirtilmiş olan Etik İlkeleri gereğince değerlendirilmiştir.

KARAR: Kırıkkale Üniversitesi Diş Hekimliği Fakültesi Ağız Diş ve Çene Radyolojisi Anabilim Dalı, Doç. Dr. Melda MISIRLIOĞLU'nun danışmanlığında yürütülecek olan Arş. Gör. Rabia ÇELİK'in uzmanlık tezi **"Dişli, Dişsiz ve Kısmi Dişli Hastalarda Maksiller Sinüs Hacmi ve Septa Prevelansının Konik Işınlı Bilgisayarlı Tomografi ile Değerlendirilmesi"** isimli başvurusu Kırıkkale Üniversitesi Girişimsel Olmayan Araştırmalar Etik Kurulu Yönergesinde belirtilmiş olan Etik İlkelere uygun bulunmuştur.

Prof.Dr. Sema ZERGEROĞLU
Başkan

Prof. Dr. Yasin DEMİRBAŞ
Üye

Doç. Dr. Mehmet Zahit ADIŞEN
Üye

Doç. Dr. Oktay AYDIN
Üye

Doç. Dr. Meral SERTEL
Üye

Doç. Dr. Funda ERDUGAN
Üye

Doç. Dr. Burak Mustafa TAŞ
Üye

Doç. Dr. Dilek AZKUR
Üye

Dr. Öğr. Üyesi Murat UYGURTAŞ
Üye
